# Measurement properties of the EQ-5D-Y-3L, PedsQL 4.0, and PROMIS-25 Profile v2.0 in pediatric patients with spinal muscular atrophy

**DOI:** 10.1186/s12955-024-02264-9

**Published:** 2024-06-27

**Authors:** Richard Huan Xu, Zuyi Zhao, Zhuxin Mao, Shengfeng Wang, Hui Xiong, Dong Dong

**Affiliations:** 1 Department of Rehabilitation Sciences, Faculty of Health and Social Sciences, Hong Kong Polytechnic University, Hong Kong, China; 2 Centre for Health Economics Research and Modelling Infectious Diseases (CHERMID), University of Antwerp, Antwerp, Belgium; 3 Department of Epidemiology and Biostatistics, School of Public Health, Peking University, Beijing, China; 4 Department of Pediatrics, Peking University First Hospital, Beijing, China; 5 JC School of Public Health and Primary Care, The Chinese University of Hong Kong, Hong Kong, China

**Keywords:** Measurement properties, EQ-5D-Y-3L, PedsQL, PROMIS-25, Spinal muscular atrophy

## Abstract

**Objective:**

The objective of this study was to examine the psychometric properties of the EQ-5D-Y-3 L, Patient Reported Outcomes Measurement System 25-item version profile v2.0 (PROMIS-25), and Pediatric Quality of Life Inventory™ version 4.0 Generic Core Scale (PedsQL 4.0) in Chinese pediatric patients with spinal muscular atrophy (SMA).

**Methods:**

The data used in this study were obtained via a web-based cross-sectional survey. Parents of pediatric patients with SMA completed the proxy-reported EQ-5D-Y-3 L, PedsQL 4.0, and PROMIS-25 measures. Information about socioeconomic and health status was also obtained. The ceiling and floor effects, factorial structure, convergent validity, and known-group validity of the three measures were assessed.

**Results:**

Three hundred and sixty-three parents of children aged from 5 to 12 completed the questionnaires. Strong floor effects were observed for the physical function components of the PROMIS-25 (41.3%) and PedsQL 4.0 (67.8%). For EQ-5D-Y-3 L, 84.6% of the respondents reported having “a lot of” problems with the dimensions “walking” and “looking after myself.” Minimal ceiling or floor effects were observed for the EQ-5D-Y-3 L index value. The confirmatory factor analysis supported a six-factor structure for the PROMIS-25, but did not support a four-factor structure for the PedsQL 4.0. All hypothesized correlations of the dimensions among the three measures were confirmed, with coefficients ranging from 0.28 to 0.68. Analysis of variance showed that EQ-5D-Y-3 L demonstrated better known-group validity than the other two measures in 14 out of 16 comparisons.

**Conclusions:**

The EQ-5D-Y-3 L showed better discriminant power than the other two measures. The physical health dimensions of all three measures showed the significant floor effects. These findings provide valuable insights into the effectiveness of these measures at capturing and quantifying the impact of SMA on patients’ health-related quality of life.

**Supplementary Information:**

The online version contains supplementary material available at 10.1186/s12955-024-02264-9.

## Introduction

Spinal muscular atrophy (SMA) is a rare debilitating neurodegenerative disorder that exhibits an autosomal recessive inheritance pattern. It manifests as the progressive degeneration of alpha motor neurons, which are situated in the spinal cord [1]. The loss of alpha motor neurons has profound implications for the intricate communication network between the central nervous system and the muscles. This disruption significantly impairs an individual’s ability to carry out basic everyday tasks. The incidence of SMA is approximately 10 in 100,000 live births, and it has three main types [2]. Type 1 SMA is the most severe form, accounting for 45% of cases. It usually develops from birth to 6 months of age. Type 2 comprises 20% of cases, and it typically develops from 6 to 18 months of age. Type 3 SMA accounts for approximately 30% of cases, and it develops from 18 months of age to adulthood. Patients with type 3 SMA can usually stand or walk independently but may experience mild weakness in their upper limbs as the disease progresses.

SMA and its treatments have either short- or long-term negative impacts on pediatric patients’ health-related quality of life (HRQoL). For instance, a study in Thailand indicated that HRQoL was significantly poorer in children with SMA than in healthy children [3]. A systematic review demonstrated that both children and adults with SMA experience impaired HRQoL [4]. Another study in China found that HRQoL was relatively lower in children with type I and type II SMA, as well as in their caregivers, compared with those with type III SMA [5]. Previous research has shown that patients with SMA often face difficulties in performing basic daily activities and may develop complications, such as joint contractures [6] and scoliosis [7], and thus their HRQoL is typically low. This reduction in physical function prevents patients from participating in social and leisure activities, further contributing to their lower HRQoL. While there is currently no cure for SMA, there are treatment options available to manage SMA symptoms and slow disease progression. For example, gene therapy [8] and movement therapy [9] have shown promising results in terms of improving motor function and enhancing the HRQoL of individuals with SMA. However, it is important to note that these treatments may have adverse consequences, such as fever, rashes, and diarrhea [10], which could also potentially worsen their HRQoL. Therefore, it is crucial to understand the impact of symptoms, medications, and side effects on reduced HRQoL in individuals with SMA to provide comprehensive care. However, currently, there are no specific measures available to assess the HRQoL of pediatric patients with SMA.

HRQoL in pediatric patients can be assessed using both preference and non-preference patient-reported outcome measures. The Pediatric Quality of Life Inventory™ version 4.0 Generic Core Scale (PedsQL 4.0) is the most frequently used non-preference measure to evaluate HRQoL in children from 2 to 18 years of age [11]. Another non-preference measure is the Patient Reported Outcomes Measurement System 25-item version profile v2.0 (PROMIS-25). It is a PROMIS-related measure specifically calibrated for children and adolescents aged 8 to 17 [12]. EQ-5D-Y-3 L is a preference-based measure that is a modified version of the original EQ-5D-3 L for adults. It has been adapted to assess HRQoL in children and adolescents aged 8 and over. The proxy version can be used for children aged from 4 to 7. In recent years, the application of these measures has been expanded to various populations and patient groups. However, none of the three measures are designed to gather disease-specific data or provide a comprehensive understanding of the factors influencing HRQoL in individuals with rare neuromuscular disorders, such as SMA. Therefore, it may not fully capture the unique challenges and subtleties associated with SMA.

SMA-specific HRQoL measures are limited. One option is the SMA Independence Scale-Upper Limb Module [13]. This measure indirectly assesses the HRQoL of patients with SMA by measuring the level of assistance that they require to perform daily activities. However, there is limited evidence supporting its psychometric performance. Consequently, a generic measure may currently be the most suitable measure to assess HRQoL in this population. The measurement properties of EQ-5D-Y-3 L, PedsQL 4.0, and PROMIS-25 have been assessed and compared in various patient groups, but not in patients with SMA. PedsQL alone has been validated in patients with SMA in some studies [14], but there is no evidence regarding its validity in the Asian patients. Additionally, our previous study demonstrated that the adult version of EQ-5D is acceptable for use in patients with SMA [11], but the performance of the children-friendly version (EQ-5D-Y-3 L) remains unknown. A recent systematic review revealed that measuring HRQoL in children with SMA poses a unique challenge. It indicated the importance of examining and comparing the effectiveness of commonly used measures in patients with all types of SMA [14]. To date, no studies have compared the measurement properties of these measures in SMA. Therefore, the objective of this study was to examine the psychometric properties of the EQ-5D-Y-3 L, PROMIS-25, and PedsQL 4.0 in a group of Chinese pediatric patients with SMA. Specifically, we evaluated the factorial structure, convergent validity, and known-group validity of these measures in this population.

## Methods

### Data and participants

The data used in this study were obtained via a web-based cross-sectional survey conducted in China from May to June 2022. The research team collaborated with a patient association (Meier Advocacy & Support Centre for SMA) to recruit individuals with SMA. The parents of pediatric patients with SMA were invited to join the survey. The parents were included if (1) they perceived themselves as a primary caregiver; (2) their child was aged from 5 to 12 years at the time of the study; (3) they had no cognitive problems; and (4) they were able to provide informed consent. Information regarding the study was sent to all of the eligible parents via the patient organization’s internal social network. Thereafter, all interested members were invited to join an online chat group, and a link to introductory information about the study and the questionnaire was shared with the group. Participants could participate in the formal survey by clicking on the link provided. All of the participants were required to complete the EQ-5D-Y-3 L, PROMIS-25, and PedsQL 4.0 questionnaires. Additional information about their sociodemographic and health status was also collected. The Institutional Review Board of the Chinese University of Hong Kong approved the study protocol and the informed consent form (Ref no.: SBRE-18-268). All of the participants provided written informed consent.

### Measures

#### EQ-5D-Y-3 L

The patient-proxy version of EQ-5D-Y-3 L was used in this study [15]. Its descriptive system has five items (walking about, looking after myself, doing usual activities, having pain or discomfort, and feeling worried, sad, or unhappy). Each item has three option levels (no problems, some problems, and a lot of problems). EQ-5D-Y-3 L also includes a visual analog scale (EQ VAS), where the respondent rates their overall health status on a scale from 0 to 100, with 0 representing the worst and 100 the best health state they can imagine. In this study, the index value of EQ-5D-Y-3 L was estimated using the Chinese value set [16], where higher scores indicate better HRQoL. The psychometric properties of EQ-5D-Y-3 L in Chinese children and adolescents have been confirmed by Wang et al. [17].

#### PROMIS-25

The patient-proxy version of the PROMIS-25 was used in this study. It consists of six HRQoL domains (mobility, anxiety, depressive symptoms, fatigue, peer relationships, and pain interference) with four questions per domain. The 25th item is a 0-to-10 numerical rating scale item for pain intensity. All other items, except for this one, are rated on a 5-point Likert scale, and the raw item scores are summed and converted to a *T*-score metric using the scoring manual [18], with higher *T*-scores indicating better HRQoL. The psychometric properties of the PROMIS-25 in the Chinese population have been reported by Li et al. [19].

#### PedsQL 4.0

The 23-item patient-proxy versions of the PedsQL 4.0 Generic Core Scale for age groups 5–7 (young child) and 8–12 (child) were used in this study [20]. It includes four domains: physical functioning (8 items), emotional functioning (5 items), social functioning (5 items), and school functioning (5 items). A 5-point response scale was used and each version having essentially identical items. The items were reverse-scored and transformed to a 0-to-100 scale, with higher scores indicating better HRQoL. The psychometric properties of the PedsQL 4.0 in the general Chinese population have been reported by Hao et al. [21].

### Statistical analysis

R software was used to perform all of the analyses [22], and the significance level was set at *p* ≤ 0.05. Descriptive analysis was used to describe the patients’ background characteristics, health status, and profile (e.g., mean, standard deviation [SD], and median) of three measures.

#### Floor and ceiling effects

The percentage of participants with the highest or lowest possible scores for each dimension of the EQ-5D-Y-3 L, PROMIS-25, and PedsQL 4.0 measures and their overall score were calculated to examine the floor and ceiling effects. Ceiling and floor effects were defined as the number and percentage of patients with the best (ceiling) and worst (floor) level of health in each dimension and across all dimensions (e.g., for EQ-5D-Y-3 L, “11111” and “33333” health states, respectively) [23]. According to the suggestion by Terwee et al. [24], ceiling and floor effects were considered problematic if 15% or more of the sample was at the ceiling or floor of the overall score.

### Factorial structure and reliability

As the items of EQ-5D-Y-3 L were designed to be independent of each other, factor analysis and the assessment of internal consistency were not appropriate for this measure. Therefore, the factorial structure and reliability were only assessed for the PROMIS-25 and PedsQL 4.0. For factor analysis, we first used confirmatory factor analysis (CFA) to assess the factorial structure of the two measures. We tested a four-factor structure for the PedsQL 4.0 and a six-factor structure for the PROMIS-25. The fit of the model was evaluated by checking the comparative fit index (CFI, > 0.9), the Tucker–Lewis index (TLI, > 0.9), the root mean square error of approximation (RMSEA, < 0.08), and the factor loading (> 0.3). If the CFA results did not support the model, we used exploratory factor analysis (EFA) to explore the factorial structure of the measures. Bartlett’s test of sphericity (*p* < 0.05, accept) and the Kaiser–Meyer–Olkin (KMO) test (> 0.6, accept) were used to validate the assumptions of the EFA. The optimal number of factors was determined through factor diagnostics, which included an eigenvalue greater than 1, a very simple structure, and parallel analysis. EFA was conducted on the polychoric correlation matrix using a promax rotation.

The internal consistency of the PROMIS-25 and PedsQL 4.0 was evaluated using Cronbach’s alpha. An alpha value greater than 0.7 was considered acceptable, a value greater than 0.8 was good, and a value greater than 0.9 was excellent [25].

### Convergent validity

Convergent validity was assessed by hypothesis testing. We assumed several correlations between the EQ-5D-Y-3 L, PROMIS-25, and PedsQL 4.0 similar domains. For example, we assumed that there is a moderate-to-strong correlation between the EQ-5D-Y-3 L dimension “walking about” and the PROMIS-25 dimension “mobility” and between the EQ-5D-Y-3 L dimension “feeling worried, sad, or unhappy” and the PedsQL 4.0 subscale “emotional functioning.” Spearman’s correlation coefficient (ρ) was used to assess the strength of the dimension-leveled correlation (≤ 0.19, very weak; 0.2–0.39, weak; 0.4–0.59, moderate; 0.6–0.79, strong; and ≥ 0.8, very strong) [26]. Pearson’s correlation coefficient (r) was used to examine the correlations between the EQ-5D-Y-3 L index values and the EQ VAS, PedsQL 4.0, and PROMIS-25 subscale scores, (*r* ≤ 0.29, weak; *r* ≤ 0.49 moderate; and *r* > 0.49 strong) [26].

### Known-group validity

The discriminatory ability of the EQ-5D-Y-3 L index value, PedsQL 4.0 overall score, and PROMIS-25 level sum score was examined using analysis of variance (ANOVA) based on the respondents’ reported clinical conditions (e.g., use of airway cleaning, scoliosis, and ability to hold up the head without support). These conditions were confirmed based on our literature review and discussions with an expert team from the patient association. We hypothesized that individuals showing clinical symptoms/conditions would likely report worse scores on HRQoL measures: lower scores on both EQ-5D-Y3L and PedsQL, while showing higher level sum score on the PROMIS-25. The F-statistic, Cohen’s D value (< 0.5 are considered small, < 0.8 moderate, and ≥ 0.8 large [27]), and/or the eta squared value (used for multi-group comparisons; small > 0.01, medium > 0.06, and large > 0.14 [28]) were used to evaluate the efficiency of the measures at differentiating patients with various clinical conditions or physical functioning.

## Results

### Respondents’ background characteristics

The demographic information for patients with SMA who took part in this study is presented in Table 1. Three hundred and sixty-three participants completed the questionnaires (response rate = 94%, 363/386). The proportions of male and female patients were similar (53.7% and 46.3%, respectively), 22% of the patients were between the ages of 5 and 7, 31.7% were enrolled in an educational institution, and the majority of diagnoses (66.1%) were type II SMA. The average duration since the diagnosis of SMA was 9.2 years. Mothers made up the majority of the patients’ caregivers (77.1%), and most of them were aged from 30 to 40.

**Table 1:** patients’ characteristics

	*N* = 363 (%)
**Patients**	
Sex	
Male	195 (53.7)
Female	168 (46.3)
Age	
5–7	80 (22)
8–12	283(78)
Whether accepting education	
Yes	115 (31.7)
No	168 (46.3)
Type	
I	45 (12.4)
II	240 (66.1)
III	78 (21.5)
Duration (year) since diagnosis, mean (SD)	9.2 (1.9)
**Caregivers**	
Parents	
Father	83 (22.9)
Mother	280 (77.1)
Age	
<30	12 (3.3)
30–40	244 (37.2)
>40	102 (28.1)

### Measurement profile

Table 2 presents the measurement profiles for the three measures. The mean (SD) index values were 0.52 (0.17) for EQ-5D-Y-3 L and 55.5 (24.6) for the EQ VAS. The mean scores of the six domains of the PROMIS-25 ranged from 24.3 (5.5) to 55.9 (9.0). The mean score of the physical functioning domain of the PedsQL 4.0 was 7.2, but more than 60% of participants reported a score of 0. The mean values for the PROMIS-25 [29] and PedsQL 4.0 [21] reported in general Chinese populations are also displayed in Table 2 for reference.

**Table 2:** statistical analysis of domains in PROMIS-25, PedsQL 4.0, and EQ-5D-Y-3 L

	Mean (SD)	Median	Min (%)	Max (%)	Theoretical range	Mean value from the other Chinese population
**PROMIS-25**						
Physical function	24.3 (5.5)	23.1	20.1 (41.3)	57.1 (0.8)	20.1–57.1	42.5(17.2)
Anxiety	53.5 (9.9)	54.8	35.6 (13.8)	79.5 (1.1)	35.6–79.5	49.5(10.9)
Depressive symptoms	53.3 (8.5)	54.6	37.7 (15.7)	78.7 (0.3)	37.7–78.7	50.2(10.2)
Fatigue	55.9 (9.0)	56.5	35.4 (7.7)	77.6 (1.7)	35.4–77.6	51.7(10.4)
Peer relationships	43.4 (8.6)	42.6	23.0 (2.2)	61.1 (9.9)	23-61.1	43.2(10.5)
Pain impact	49.9 (10.2)	49.3	36.7 (28.9)	67.7 (1.1)	36.7–74	49.3(10.1)
Pain Intensity	1.9 (2.0)	1.0	0 (31.1)	10 (0.3)	0–10	2.8(2.7)
Level sum score	58.9 (17.7)	59	24(0.3)	88(0.3)	25–125	-
**PedsQL 4.0**						
Physical functioning score	7.2 (16.8)	0	0.0 (67.8)	75.0 (3.3)	0-100	87.3(9.8)
Emotional functioning score	68.4 (19.2)	65	0.0 (0.6)	100.0 (15.4)	0-100	83.0(15)
Social functioning score	51.3 (19.0)	50	0.0 (1.1)	100.0 (3.9)	0-100	90.0(12.9)
School functioning score	67.3 (16.7)	65	20.0 (0.3)	100.0 (3.0)	0-100	85.2(13.3)
Overall score	29.8 (13.9)	28.3	0(0.6)	87.5(0.6)	0-100	-
**EQ-5D-Y-3 L**						
Index value	0.52 (0.17)	0.51	-0.09 (1.1)	0.99 (1.1)	-0.088-1	-
EQ VAS score	55.5 (24.6)	60	0.0 (1.4)	100.0 (2.8)	0-100	-

### Ceiling and floor effects

**Fig. 1:**
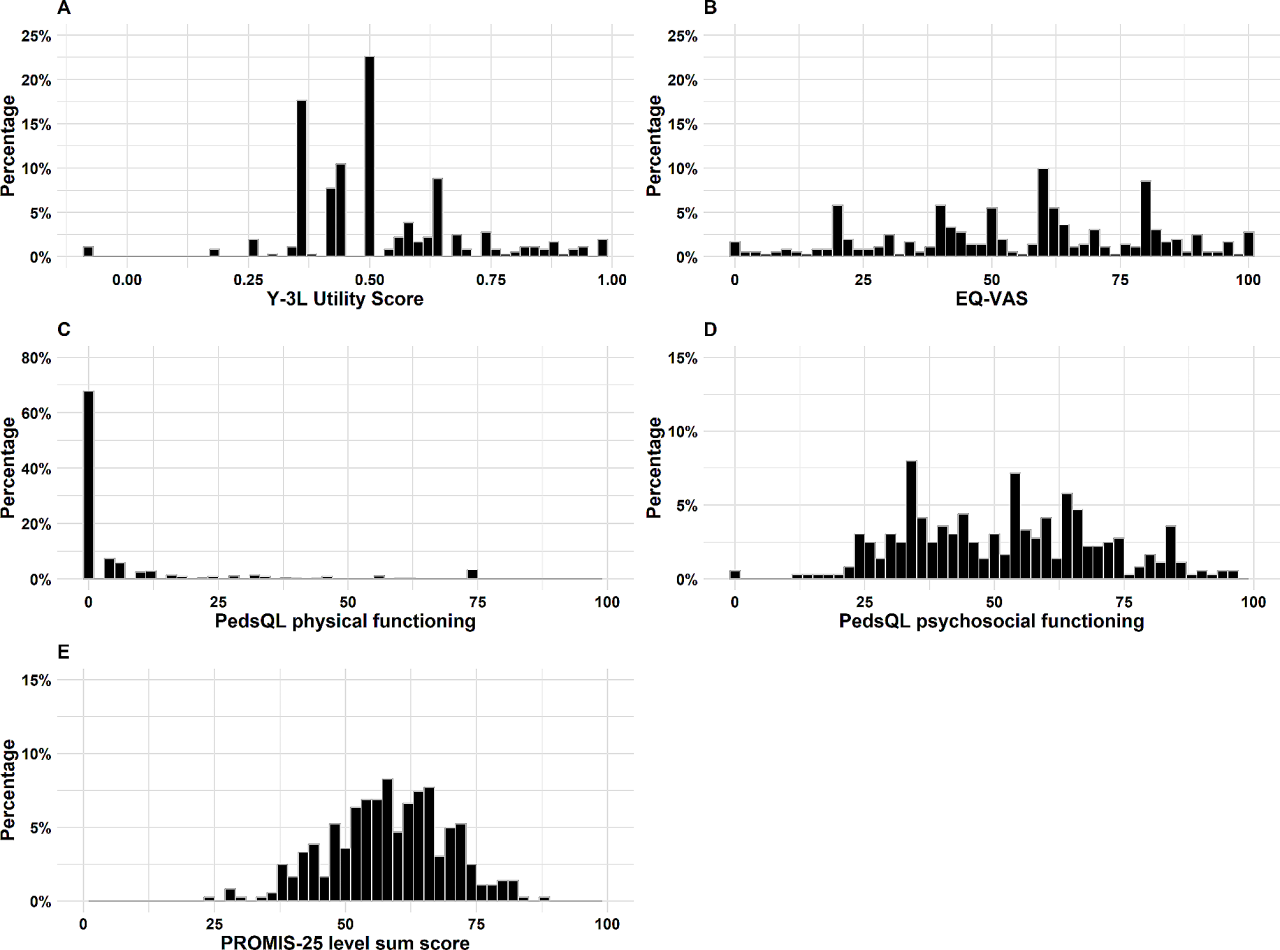
the score distribution for three measures

The distributions of the responses to the dimensions or items of the three measures are presented in Table 3. For the PROMIS-25, a high proportion of participants indicated they were “not able to do” the activities in the “mobility” domain. The percentages ranged from 44.1 to 92%, which were higher than the percentages for the other five domains of PROMIS-25. However, the level sum score of the PROMIS-25 was evenly distributed, showing a unimodal distribution and concentrating around 55 (Fig. 1E). For the PedsQL 4.0, a high proportion of participants selected the worst option in six out of the eight items related to physical functioning, with 73.3–92.3% selecting “almost always.” For the other three domains, most of the items showed a high proportion of patients selecting the best option, ranging from 18.6% (“missing school because of not feeling well” in the school functioning domain) to 37.3% (“forgetting things” in the school functioning domain). For EQ-5D-Y-3 L, 56.7% of the respondents reported “no problem” for the dimension of “having pain or discomfort.” Approximately 47.7% of the respondents reported “no problem” for the dimension of “feeling worried, sad, or unhappy.” Additionally, approximately 84.6%, 84.6%, and 62% of the respondents reported having “a lot of problems” for the dimensions of “walking about,” “looking after myself,” and “doing usual activities,” respectively. Regarding scale-level, minimal ceiling effects were observed for the EQ-5D-Y-3 L (1.1%).

**Table 3:** Percentage of reported problems and summarized scores of the PROMIS-25, PedsQL 4.0, and EQ-5D-Y-3 L

	*N*(%)				
	**Level 1**	**Level 2**	**Level 3**	**Level 4**	**Level 5**
**PROMIS-25**					
**Mobility**					
Do sports and exercises	7 (1.9)	2 (0.6)	24 (6.6)	44 (12.1)	286 (78.8)
Get up from the floor	7 (1.9)	3 (0.8)	23 (6.3)	29 (8.0)	301 (82.9)
Walk up stairs	5 (1.4)	3 (0.8)	8 (2.2)	13(3.6)	334 (92)
Do activities they enjoy	26 (7.2)	23 (6.3)	76 (20.9)	78 (21.5)	160 (44.1)
**Anxiety**					
Felt awful things would happen	88(24.2)	107 (29.5)	27 (7.3)	134 (36.9)	7 (1.9)
Felt nervous	71 (19.6)	96 (26.4)	26 (7.2)	162 (44.6)	8 (2.2)
Felt worried	71 (19.6)	89 (24.5)	30 (8.3)	161 (44.4)	12 (3.3)
Worried when at home	75 (20.7)	97 (26.7)	33 (9.1)	138 (38.0)	20 (5.5)
**Depressive Symptoms**					
Felt everything went wrong	114 (31.4)	135 (37.2)	11 (3.0)	101 (27.8)	2 (0.6)
Felt lonely	72 (19.8)	111 (30.6)	31 (8.5)	138 (38.0)	11 (3.0)
Felt sad	84 (23.1)	120 (33.1)	16 (4.4)	140 (38.6)	3 (0.8)
Hard to have fun	92 (25.3)	115 (31.7)	14 (3.9)	138 (38.0)	4 (1.1)
**Fatigue**					
Hard to keep up with schoolwork	67 (18.5)	105 (28.9)	28 (7.7)	129 (35.5)	34 (9.4)
Got tired easily	44 (12.1)	73 (20.1)	61 (16.8)	164 (45.2)	21 (5.8)
Tired to sports	35 (9.6)	64 (17.6)	55 (15.2)	139 (38.3)	70 (19.3)
Tired to enjoy things they like	65 (17.9)	129 (35.5)	18 (5.0)	138 (38.0)	13 (3.6)
**Peer Relationships**					
Felt accepted by other kids	18 (5.0)	32 (8.8)	114 (31.4)	113 (31.1)	86 (23.7)
Counted on friends	21 (5.8)	73 (20.1)	99 (27.3)	120 (33.1)	50 (13.8)
Helped with friends each other	15 (4.1)	54 (14.9)	116 (32.0)	112 (30.9)	66 (18.2)
Other kids wanted to be their friends	12 (3.3)	33 (9.1)	111 (30.6)	128 (35.3)	79 (21.8)
**Pain Impact**					
Hard to fall asleep	151 (41.6)	139 (38.3)	7 (1.9)	65 (17.9)	1 (0.3)
Hard to concentrated	149 (41.0)	133 (36.6)	4 (1.1)	77 (21.2)	0
Hard to run	145 (39.9)	62 (17.1)	11 (3.0)	11 (3.0)	134 (36.9)
Hard to walk 100 m	147 (40.5)	51 (14.0)	7 (1.9)	20 (5.5)	138 (38.0)
**PedsQL 4.0**	**Level 1**	**Level 2**	**Level 3**	**Level 4**	**Level 5**
**Physical Functioning**					
Walking more than one block	29 (8.0)	4 (1.1)	16 (4.4)	11 (3.0)	303 (83.5)
Running	14 (3.8)	4 (1.1)	4 (1.1)	6 (1.7)	335 (92.3)
Participating in sports	13 (3.6)	7 (2.0)	11 (3.0)	11 (3.0)	321 (88.4)
Lifting heavy thing	14 (3.8)	6 (1.7)	13 (3.6)	25 (6.9)	305 (84.0)
Taking a bath or shower by him/herself	18 (5.0)	8 (2.2)	16 (4.4)	23 (6.3)	298 (82.1)
Doing chores around the house	16 (4.4)	9 (2.5)	33 (9.1)	39 (10.7)	266 (73.3)
Having hurts or aches	131 (36.1)	102 (28.1)	106 (29.2)	18 (5.0)	11 (3.0)
Low energy level	69 (19.0)	69 (19.0)	171 (47.1)	43 (11.9)	11 (3.0)
**Emotional Functioning**					
Feeling afraid/ scared	90 (24.7)	95 (26.2)	157 (43.3)	18 (5.0)	3 (0.8)
Feeling sad or blue	87 (24.0)	114 (31.4)	152 (41.9)	7 (1.9)	3 (0.8)
Feeling angry	69 (19.0)	92 (25.3)	181 (49.9)	18 (5.0)	3 (0.8)
Trouble sleeping	105 (28.9)	135 (37.2)	99 (27.3)	19 (5.2)	5 (1.4)
Worrying about what will happen to him or her	96 (26.4)	124 (34.2)	116 (32.0)	23 (6.3)	4 (1.1)
**Social Functioning**					
Getting along with other children	108 (29.8)	126 (34.7)	107 (29.5)	11 (3.0)	11 (3.0)
Other kids not wanting to be his or her friend	99 (27.3)	131 (36.1)	105 (28.9)	18 (5.0)	10 (2.7)
Getting teased by other children	89 (24.5)	123 (33.9)	131 (36.1)	13 (3.6)	7 (1.9)
Not able to do things that other children can do	22 (6.1)	11 (3.0)	63 (17.4)	78 (21.5)	189 (52.0)
Keeping up when playing with other children	20 (5.5)	14 (3.9)	68 (18.7)	91 (25.1)	170 (46.8)
**School Functioning**					
Paying attention in class	67 (32.8)	63 (30.9)	62 (30.4)	11 (5.4)	1 (0.5)
Forgetting things	76 (37.3)	71 (34.8)	48 (23.5)	9 (4.4)	0 (0)
Keeping up with schoolwork	70 (34.3)	63 (30.9)	48 (23.5)	13 (6.4)	10 (4.9)
Missing school because of not feeling well	38 (18.6)	55 (20.7)	90 (44.1)	16 (7.8)	5 (2.5)
Missing school to go to the doctor or hospital	19 (9.3)	30 (14.7)	124 (60.8)	26 (12.7)	5 (2.5)
**EQ-5D-Y-3 L**	**Level 1**	**Level 2**	**Level 3**	-	-
Walking about	20 (5.5)	36 (9.9)	307 (84.6)	-	-
Looking after myself	11 (3.0)	45 (12.4)	307 (84.6)	-	-
Doing usual activities	32 (8.8)	106 (29.2)	225 (62.0)	-	-
Having pain or discomfort	206 (56.7)	148 (40.8)	9 (2.5)	-	-
Feeling worried, sad or unhappy	173 (47.7)	174 (47.9)	16 (4.4)	-	-
Full health status (11,111)	4 (1.1)				

Note: PROMIS-25, the five option levels: no trouble (never), a little trouble (almost never), some trouble (sometimes), a lot of trouble (often),

and not able to do (almost always)

PedsQL 4.0, the five option levels: never, almost never, sometimes, often, almost always

For EQ-5D-Y-3 L, the five option levels: no problem, some problem, and a lot of problem

### Factorial structure

Initially, two CFA models were developed to assess the factorial structure of the three measures separately (Appendix, Table A1). The results showed that the six-factor model of the PROMIS-25 was supported, with an RMSEA value of 0.057, a CFI value of 0.956, and a TLI value of 0.948. However, the four-factor model of the PedsQL 4.0 was not supported, consequently, EFA was conducted to explore its factorial structure. Table 4 presents the results of the EFA. The KMO value for PedsQL 4.0 was 0.88, indicating acceptable sampling adequacy, and Bartlett’s test of sphericity was significant (*p* < 0.001), supporting the factorability of the data. A four-factor structure was determined for the PedsQL 4.0, but the item distribution did not align with expectations. Only the first six items were included in the physical functioning factor, while items 7 and 8 were included in the emotional functioning factor. The social functioning factor is represented by only one item, and the factor loadings for the other four items were less than 0.3. The Cronbach’s alpha coefficients indicated good internal consistency for the PROMIS-25 (0.81) and PedsQL 4.0 (0.89).

**Table 4:** Exploratory factor analysis and reliability of three measures

PROMIS-25^a^ 6-factor model			PedsQL 4.0 4-factor model		
Do sports and exercises	F1	0.844	Walking more than one block	F1	0.824
Get up from the floor		0.855	Running		0.921
Walk up stairs		0.856	Participating in sports		0.97
Do activities they enjoy		0.443	Lifting heavy thing		0.965
Felt awful things would happen	F2	0.853	Taking a bath or shower by him/herself		0.91
Felt nervous		0.895	Doing chores around the house		0.848
Felt worried		0.941	Having hurts or aches	F2	0.645
Worried when at home		0.767	Low energy level		0.566
Felt everything went wrong	F3	0.765	Feeling afraid/ scared		0.882
Felt lonely		0.791	Feeling sad or blue		0.923
Felt sad		0.881	Feeling angry		0.825
Hard to have fun		0.825	Trouble sleeping		0.733
Hard to keep up with schoolwork	F4	0.762	Worrying about what will happen to him or her		0.784
Got tired easily		0.840	Getting along with other children	F3	0.42
Tired to sports		0.762	Other kids not wanting to be his or her friend		*< 0.3*
Tired to enjoy things they like		0.773	Getting teased by other children		*< 0.3*
Felt accepted by other kids	F5	0.734	Not able to do things that other children can do		*< 0.3*
Counted on friends		0.857	Keeping up when playing with other children		*< 0.3*
Helped with friends each other		0.919	Paying attention in class	F4	0.833
Other kids wanted to be their friends		0.818	Forgetting things		0.852
Hard to fall asleep	F6	0.910	Keeping up with schoolwork		0.802
Hard to concentrated		0.942	Missing school because of not feeling well		0.706
Hard to run		0.513	Missing school to go to the doctor or hospital		0.97
Hard to walk 100 m		0.508			-
**Cronbach’s alpha**		0.81			0.89

a. for PROMIS-25, the CFA factor loadings are presented

### Convergent validity

All 13 pairs of similar dimensions from the three measures, PROMIS-25, PedsQL 4.0, and EQ-5D-Y-3 L, exhibited statistically significant correlations, confirming the convergent validity (Table 5). Among these, four pairs showed strong correlations (|ρ| = 0.61–0.68). Two of these pairs correlated “walking about” on EQ-5D-Y-3 L with “mobility” on the PROMIS-25 and “physical functioning” on the PedsQL 4.0. The other two pairs correlated “emotional functioning” on the PedsQL 4.0 with “anxiety” and “depressive symptoms” on the PROMIS-25. Seven pairs showed moderate correlations (|ρ| = 0.46–0.57), while two pairs exhibited relatively weak correlations (|ρ| = 0.28–0.33). Additionally, the EQ-5D-Y-3 L index value demonstrated a stronger correlation than the EQ VAS score, with the overall score for the PedsQL 4.0 (|r| = 0.56) and the level sum scores of the PROMIS-25 (|r| = 0.26). A moderate correlation was observed between the overall score for the PedsQL 4.0 and the level sum score for the PROMIS-25 (|r| = 0.32). The correlations between dimensions for all comparisons among the three measures are presented in the Appendix (Table A2).

**Table 5:** Correlations of similar dimensions/overall scores between measures

Dimension	Dimension	ρ / *r*^a^	*p*-value
**EQ-5D-Y-3 L**	**PROMIS-25**		
Walking about	Mobility	-0.61	< 0.001
Having pain or discomfort	Pain interference	0.48	< 0.001
Feeling worried, sad, or unhappy	Depressive symptoms	0.57	< 0.001
Feeling worried, sad, or unhappy	Anxiety	0.53	< 0.001
**EQ-5D-Y-3 L**	**PedsQL 4.0**		
Walking about	Physical functioning	-0.61	< 0.001
Looking after myself	Physical functioning	-0.51	< 0.001
Doing usual activities	Social functioning	-0.33	< 0.001
Feeling worried, sad, or unhappy	Emotional functioning	-0.55	< 0.001
**PedsQL 4.0**	**PROMIS-25**		
Physical functioning	Mobility	0.52	< 0.001
Emotional functioning	Anxiety	-0.68	< 0.001
Emotional functioning	Depressive symptoms	-0.67	< 0.001
Social functioning	Peer relationships	0.46	< 0.001
School functioning	Peer relationships	0.28	< 0.001
**EQ-5D-Y-3 L**	**PedsQL 4.0**		
Index value	Overall score	0.56	< 0.001
EQ VAS	Overall score	0.25	< 0.001
**EQ-5D-Y-3 L**	**EQ-5D-Y-3 L**		
Index value	EQ VAS	0.34	< 0.001
**EQ-5D-Y-3 L**	**PROMIS-25**		
Index value	Level sum score	-0.26	< 0.001
EQ VAS	Level sum score	-0.2	< 0.001
**PedsQL 4.0**	**PROMIS-25**		
Overall score	Level sum score	-0.32	< 0.001

a. For the comparisons between dimensions, the ρ was reported. For the comparisons between index value or overall scores, the r was reported

### Known-group validity

The EQ-5D-Y-3 L index value, EQ VAS score, and PedsQL 4.0 overall score were able to differentiate HRQoL across different symptom or condition groups (Table 6). The EQ-5D-Y-3 L index value and the PedsQL 4.0 overall score demonstrated satisfactory known-group validity, as they were sensitive at identifying different levels of HRQoL for all comparisons. However, based on the F-statistics, Cohen’s D or Eta-squared values, EQ-5D-Y-3 L demonstrated stronger discriminant ability than the other measures. This was particularly true for standing and walking, which are two essential physical functions for patients with SMA. The F-statistics value for the EQ-5D-Y-3 L index value was approximately three times larger than that of the PedsQL 4.0 overall score for “standing alone without support.” Similarly, for “walking alone without support,” the F-statistics value of the EQ-5D-Y-3 L index value was approximately four times larger than that of the PedsQL 4.0 overall score and approximately 20 times larger than that of the EQ VAS score. However, the PROMIS-25 level sum score did not identify statistically significant differences in HRQoL between any symptom or condition groups, indicating poor known-group validity.

**Table 6:** known-group validity of the EQ-5D-Y-3 L, PROMIS-25, and PedsQL

	*N*	EQ-5D-Y-3 L Index value	EQ VAS	PedsQL 4.0 Overall score	PROMIS-25 Level sum score
**Mobile ability due to the use of wheelchair**					
No	94	0.57 (0.18)	56.6 (22.4)	32.2 (17.5)	57.8 (10.7)
Use some time	118	0.50 (0.13)	54.9 (22.4)	30.0 (11.8)	59.1 (10.8)
Use all the time	127	0.45 (0.13)	53.3 (27.3)	28.7 (12.3)	59.0 (12.0)
F-statistic		18.45	0.5	5.6	0.4
p-value		< 0.001	0.6	0.004	0.65
Eta squared		0.1	0.003	0.03	0.002
**Use of airway cleaning**					
No	274	0.54 (0.18)	56.7 (24.2)	31.7 (15.0)	58.8 (11.0)
Yes	89	0.46 (0.14)	51.8 (25.8)	24.2 (11.9)	59.0 (12.0)
F-statistic		16.91	2.6	18.1	0.02
p-value		< 0.001	0.12	< 0.001	0.89
Cohen’s D		0.5	0.2	0.52	0.02
**Scoliosis**					
No	64	0.64 (0.20)	64.4 (23.3)	39.0 (16.7)	29.1 (10.3)
Yes	213	0.49 (0.15)	53.2 (24.4)	27.2 (14.5)	58.3 (11.6)
F-statistic		20.03	5.7	18.3	2.5
p-value		< 0.001	0.004	< 0.001	0.09
Cohen’s D		0.91	0.46	0.86	0.08
**Hold up head without support**					
No	41	0.45 (0.13)	39.9 (24.2)	22.4 (13.1)	58.5 (12.7)
Yes	322	0.53 (0.17)	57.5 (24.0)	30.8 (14.6)	58.9 (11.0)
F-statistic		8.72	19.5	12.4	0.05
p-value		0.003	< 0.001	0.02	0.82
Cohen’s D		0.49	0.73	0.58	0.04
**Roll over to lateral position**					
No	176	0.45 (0.11)	51.0 (25.3)	24.5 (12.1)	58.7 (12.1)
Yes	187	0.59 (0.19)	59.7 (23.3)	34.8 (15.1)	59.1 (10.3)
F-statistic		68.21	11.6	51.1	0.1
p-value		< 0.001	0.001	< 0.001	0.71
Cohen’s D		0.87	0.36	0.75	0.04
**Sit up without support**					
No	130	0.44 (0.12)	48.6 (26.1)	23.3 (12.0)	58.2 (11.8)
Yes	233	0.57 (0.18)	59.3 (22.9)	33.5 (14.7)	59.3 (10.9)
F-statistic		47.56	16.5	45.0	0.7
p-value		< 0.001	< 0.001	< 0.001	0.41
Cohen’s D		1.67	0.44	1.18	0.06
**Four-point crawl**					
No	291	0.48 (0.13)	53.4 (24.7)	26.7 (12.1)	58.8 (11.4)
Yes	72	0.72 (0.19)	64.0 (17.7)	42.4 (17.1)	59.4 (10.5)
F-statistic		161.35	11.0	80.6	0.2
p-value		< 0.001	0.001	< 0.001	0.66
Cohen’s D					
**Stand up without support**					
No	262	0.46 (0.12)	53.3 (25.6)	26.3 (12.5)	58.9 (11.6)
Yes	101	0.68 (0.20)	61.2 (21.1)	39.0 (15.8)	58.9 (10.2)
F-statistic		174.4	7.6	63.8	0.001
p-value		< 0.001	0.006	< 0.001	0.97
Cohen’s D		1.55	0.32	0.94	0.08
**Stand alone without support**					
No	286	0.47 (0.12)	53.4 (25.1)	26.7 (12.7)	58.6 (11.5)
Yes	77	0.74 (0.16)	63.3 (21.3)	41.6 (15.4)	60.2 (10.0)
F-statistic		252.81	10.2	75.8	1.2
p-value		< 0.001	0.002	< 0.001	0.27
Cohen’s D		2.04	0.41	0.51	0.05
**Walk without support**					
No	278	0.46 (0.12)	53.4 (25.4)	26.7 (12.8)	58.8 (11.5)
Yes	85	0.72 (0.18)	62.2 (20.7)	40.1 (15.6)	59.2 (10.2)
F-statistic		220.66	8.3	64.2	0.1
p-value		< 0.001	0.004	< 0.001	0.75
Cohen’s D		1.84	0.36	0.44	0.05
**Walk alone without support**					
No	297	0.47 (0.13)	53.5 (24.9)	27.0 (12.7)	28.6 (11.4)
Yes	66	0.77 (0.15)	64.6 (21.2)	42.7(16.0)	60.4 (10.4)
F-statistic		278.8	11.3	75.0	1.5
p-value		< 0.001	0.001	< 0.001	0.22
Cohen’s D		2.27	0.46	0.57	0.06
**Walk 10 m on their own**					
No	304	0.48 (0.13)	53.7 (24.9)	27.1 (12.7)	58.5 (11.4)
Yes	59	0.77 (0.16)	64.9 (20.6)	43.8 (16.0)	60.8 (10.2)
F-statistic		234.61	10.6	77.4	2.0
p-value		< 0.001	0.001	< 0.001	0.15
Cohen’s D		2.18	0.46	0.55	0.09
**Go upstairs independently**					
No	334	0.50 (0.15)	54.5 (24.6)	28.2 (12.9)	59.0 (11.3)
Yes	29	0.81 (0.19)	67.2 (22.2)	48.5 (19.9)	58.2 (9.8)
F-statistic		109.47	7.3	59.8	0.1
p-value		< 0.001	0.007	< 0.001	0.72
Cohen’s D		2.03	0.52	0.94	0.22
**have bimanual useful function**					
No	73	0.42 (0.14)	45.8 (27.4)	26.0 (13.5)	59.5 (11.7)
Yes	290	0.55 (0.17)	57.9 (21.3)	30.8 (14.8)	58,7 (11.1)
F-statistic		35.43	14.7	6.3	0.3
p-value		< 0.001	< 0.001	0.01	0.58
Cohen’s D		0.78	0.5	0.18	0.11
**Raise hands over head when sitting**					
No	218	0.46 (0.13)	51.6 (25.7)	24.7 (11.8)	59.3 (11.8)
Yes	145	0.63 (0.18)	61.3 (21.7)	37.5 (15.2)	58.3 (10.2)
F-statistic		108.38	14.0	80.5	0.8
p-value		< 0.001	< 0.001	< 0.001	0.38
Cohen’s D		1.12	0.4	0.47	0.15
**Touch mouths while sitting**					
No	65	0.45 (0.13)	47.7 (27.6)	23.6 (11.4)	56.8 (13.1)
Yes	298	0.54 (0.18)	57.2 (23.7)	31.2 (14.9)	59.3 (10.7)
F-statistic		14.90	8.1	14.7	2.7
p-value		< 0.001	0.005	< 0.001	0.10
Cohen’s D		0.53	0.19	0.39	0.03

## Discussion

This study examined the measurement properties of three measures for assessing HRQoL in pediatric patients with SMA and compared their performance from the perspective of the primary caregivers. To the best of our knowledge, this is the first study to compare the psychometric properties of EQ-5D-Y-3 L, PedsQL 4.0, and PROMIS-25 using the same sample of patients with SMA. Overall, the findings demonstrated that EQ-5D-Y-3 L surpassed the other two measures in many aspects of psychometric properties. EQ-5D-Y-3 L showed minimal ceiling effects (1.1% for full health status) at the scale level. However, a large proportion of participants reported “no problem” for the symptom-related dimensions, but “a lot of problems” for the physical-health-related dimensions of EQ-5D-Y-3 L. This suggests a potential benefit of using the updated EQ-5D-Y-5 L version in the SMA population. Moreover, EQ-5D-Y-3 L showed higher sensitivity than both the PROMIS-25 and PedsQL 4.0 at differentiating the clinical conditions and symptoms of SMA among risk groups. This indicates that EQ-5D-Y-3 L, a brief preference-based measure, may be better suited for evaluating the effectiveness of clinical interventions in this population.

The factorial structure of the PedsQL 4.0 was not supported by CFA in this study. There were two issues with the factorial structure of the PedsQL 4.0. First, the EFA identified the last two items of the physical functioning domain (“having hurts or aches” and “low energy level”) as belonging to the emotional functioning domain. This may be due to a language issue. The Chinese expression of these two physical functioning items (“感到疼痛” and “感到疲劳”) is more consistent with the expression of an emotional functioning item (e.g., “感到悲伤,” “feeling sad or blue”). The structure of all the questions was “felt something.” Second, the factor loadings of four of the five items of social functioning were very small and could not be identified by the model. This may be because children with SMA typically have mobility problems and have fewer chances to play with peers. However, the four items of social functioning with low factor loadings all focus on the relationship with other playmates, which are not suitable items for these children [30].

Our findings showed a significant proportion of selections for the worst option in dimensions or items related to physical health. These dimensions were found to be prevalent across all three measures. In contrast, most dimensions linked to emotional problems exhibited a high proportion of selection for the best option. Similar to previous studies, the impact of SMA on physical health was evident, as affected individuals experience limitations in motor function [31] and the progressive loss of muscle strength [32]. Currently, the use of EQ-5D-Y-3 L is limited in patients with SMA, but our findings align with those of previous studies. For instance, Hu et al. found that 76.4%, 71.2%, and 68.7% of patients with SMA or their caregivers reported extreme problems with “mobility,” “looking after myself,” and “doing usual activities” [33]. Despite the high proportion of selections for the worst option for the physical health dimension across all three measures, such effects were not observed for the overall scores. This is consistent with previous findings where lower ceiling effects for the EQ-5D-Y-3 L index value were observed within certain patient groups [23]. However, for the PROMIS-25 and PedsQL 4.0, it is recommended to report dimension and sub-scale scores, respectively, rather than overall scores. This suggests that EQ-5D-Y-3 L performed better in the scale-level comparison. Nevertheless, efforts to develop index values for the PedsQL 4.0 and PROMIS-25 measures have been reported. Future studies should compare the utility level scores of the three measures.

All hypothesized correlations between the EQ-5D-Y-3 L, PedsQL 4.0, and PROMIS-25 were statistically significant, confirming their convergent validity. However, the correlations between dimensions were stronger than those of the utility or overall scores of the three measures. One possible reason is that unlike the other two non-preference measures, EQ-5D-Y-3 L is a preference-based measure and does not generate a summative score. This difference may introduce uncertainties when conducting the correlation analysis with the overall scores of the other two measures. Until now, a direct comparison between EQ-5D-Y-3 L and the other two measures has not been reported in the SMA population. Our results showed a strong association between the physical health dimensions of the three measures. This finding aligns with a recent systematic review that focused on adult patients with SMA, which suggests that SMA primarily impacts an individual’s physical HRQoL [4]. Furthermore, we identified a stronger association between emotional dimensions of the PedsQL 4.0 and PROMIS-25 than between those dimensions and the EQ-5D-Y-3 L dimension “feeling worried, sad, or unhappy.” This may be explained by the high proportion of selections for the best option in the emotional dimension of EQ-5D-Y-3 L compared with the items of the other two measures. Previous findings have been mixed, although most studies have reported similar findings to ours, indicating that EQ-5D-Y-3 L may not be sufficiently sensitive to detect differences in mental health status [34–36]. Another study found a stronger correlation between the dimension “feeling worried, sad, or unhappy” of EQ-5D-Y-3 L and the emotional dimension of the PedsQL 4.0, compared with the physical functioning dimension, in patients with osteogenesis imperfecta [37].

Our findings indicated that the EQ-5D-Y-3 L index value and the EQ VAS score, as well as the PedsQL 4.0 overall score, are sensitive at detecting differences between patients with and without clinical conditions/physical functioning related to SMA. These two measures were able to detect subtle variations in health outcomes and functioning among the groups. These results are consistent with those of previous studies [38, 39]. While the PROMIS-25 has been used for various illnesses [40, 41], we found that its discriminant power was significantly lower than that of the other two measures. This may be due to the use of the sum level score of the PROMIS-25, rather than domain scores. To ensure comparability with EQ-5D-Y-3 L and the PedsQL 4.0, we further assessed the known-group validity of the PROMIS-25 based on domain scores (Appendix, Table A3), which significantly improved the discriminant ability of the PROMIS-25.

There are several limitations of our study that need to be addressed. First, the three measures were presented to the respondents in the same order via an online survey. This may have introduced fatigue bias, potentially decreasing the reliability of our findings. Second, the responsiveness of patient-reported outcome measures is crucial in clinical practice. However, we did not examine the responsiveness of such measures, which potentially weakens the strength of our conclusions. Future studies should investigate this measurement property. Finally, while online surveys offer numerous advantages over face-to-face surveys, the data quality may not be entirely guaranteed. Participants may not be fully engaged in a long survey, which may affect the reliability of the findings.

## Conclusions

This study compared the properties of the EQ-5D-Y-3 L, PedsQL 4.0, and PROMIS-25 measures for measuring HRQoL in Chinese pediatric patients with SMA. EQ-5D-Y-3 L showed better discriminative power to distinguish HRQoL differences than the other two measures. A high proportion of participants selected the worst options for the physical health dimensions of all three measures. These findings provide valuable insights into how effectively these measures capture and measure the impact of SMA on patients’ HRQoL. Healthcare professionals should select the measure that best aligns with the unique objectives of their interventions to meet the needs of individuals affected by SMA.

### Electronic supplementary material

Below is the link to the electronic supplementary material.

Supplementary Material 1

## Data Availability

No datasets were generated or analysed during the current study.
