# Supplementary material for: Measurement properties of the EQ-5D-Y-3L, PedsQL 4.0, and PROMIS-25 Profile v2.0 in pediatric patients with spinal muscular atrophy
Source: Health Qual Life Outcomes. 2024 Jun 27;22:50. doi: 10.1186/s12955-024-02264-9 (PMC11210123; doi:10.1186/s12955-024-02264-9)
Supplement: Supplementary file 1 — Supplementary Material 1 [file 12955_2024_2264_MOESM1_ESM.docx]

**Table A1 the results of CFA**

| **PROMIS-25**  **6-factor model** | | | **PedsQL**  **4-factor model** | | |
| --- | --- | --- | --- | --- | --- |
| Do sports and exercises | F1 | 0.844 | Walking more than one block | F1 | 0.792 |
| Get up from the floor |  | 0.855 | Running |  | 0.793 |
| Walk up stairs |  | 0.856 | Participating in sports |  | 0.893 |
| Do activities they enjoy |  | 0.443 | Lifting heavy thing |  | 0.918 |
| Felt awful things would happen | F2 | 0.853 | Taking a bath or shower by him/herself |  | 0.885 |
| Felt nervous |  | 0.895 | Doing chores around the house |  | 0.833 |
| Felt worried |  | 0.941 | Having hurts or aches | F2 | 0.138 |
| Worried when at home |  | 0.767 | Low energy level |  | 0.153 |
| Felt everything went wrong | F3 | 0.765 | Feeling afraid/ scared |  | 0.817 |
| Felt lonely |  | 0.791 | Feeling sad or blue |  | 0.854 |
| Felt sad |  | 0.881 | Feeling angry |  | 0.849 |
| Hard to have fun |  | 0.825 | Trouble sleeping |  | 0.609 |
| Hard to keep up with schoolwork | F4 | 0.762 | Worrying about what will happen to him or her |  | 0.827 |
| Got tired easily |  | 0.840 | Getting along with other children | F3 | 0.822 |
| Tired to sports |  | 0.762 | Other kids not wanting to be his or her friend |  | *0.811* |
| Tired to enjoy things they like |  | 0.773 | Getting teased by other children |  | *0.665* |
| Felt accepted by other kids | F5 | 0.734 | Not able to do things that other children can do |  | *0.291* |
| Counted on friends |  | 0.857 | Keeping up when playing with other children |  | *0.362* |
| Helped with friends each other |  | 0.919 | Paying attention in class | F4 | 0.810 |
| Other kids wanted to be their friends |  | 0.818 | Forgetting things |  | 0.842 |
| Hard to fall asleep | F6 | 0.910 | Keeping up with schoolwork |  | 0.702 |
| Hard to concentrated |  | 0.942 | Missing school because of not feeling well |  | 0.276 |
| Hard to run |  | 0.513 | Missing school to go to the doctor or hospital |  | 0.296 |
| Hard to walk 100m |  | 0.508 |  |  | - |
|  |  |  |  |  |  |
| **RMSEA** |  | 0.067 |  |  | 0.114 |
| **CFI** |  | 0.956 |  |  | 0.795 |
| **TLI** |  | 0.948 |  |  | 0.768 |

Table A2 full correlations among three measures

|  | **PedsQL** | | | | **PROMIS-25** | | | | | |
| --- | --- | --- | --- | --- | --- | --- | --- | --- | --- | --- |
|  | Physical functioning | Emotional functioning | Social functioning | School functioning | Physical function | Anxiety | Depressive symptoms | Fatigue | Peer relationships | Pain interference |
| **EQ-5D-Y-3L** |  |  |  |  |  |  |  |  |  |  |
| Walking about | -0.61  (p<0.001) | 0.12  (p=0.025) | 0.18  (p=0.001) | 0.12  (p=0.078) | -0.46  (p<0.001) | 0.07  (p=0.21) | 0.06  (p=0.28) | 0.11  (p=0.03) | -0.14  (p=0.007) | 0.14  (p=0.007) |
| Looking after myself | -0.59  (p<0.001) | 0.15  (p=0.004) | 0.19  (p<0.001) | 0.14  (p=0.054) | -0.48  (p<0.001) | 0.08  (p=0.12) | 0.09  (p=0.09) | 0.15  (p=0.006) | -0.15  (p=0.005) | 0.14  (p=0.01) |
| Doing usual activities | -0.41  (p<0.001) | 0.19  (p<0.001) | 0.33  (p<0.001) | 0.13  (p=0.074) | -0.55  (p<0.001) | 0.16  (p=0.003) | 0.19  (p<0.001) | 0.26  (p<0.001) | -0.2  (p<0.001) | 0.17  (p=0.002) |
| Having pain or discomfort | -0.17  (p=0.001) | 0.32  (p<0.001) | 0.26  (p<0.001) | 0.14  (p=0.053) | -0.21  (p<0.001) | 0.3  (p<0.001) | 0.24  (p<0.001) | 0.28  (p<0.001) | -0.05  (p=0.315) | 0.48  (p<0.001) |
| Feeling worried, sad or unhappy | -0.75  (p=0.152) | 0.55  (p<0.001) | 0.37  (p<0.001) | 0.27  (p<0.001) | -0.15  (p=0.005) | 0.53  (p<0.001) | 0.57  (p<0.001) | 0.34  (p<0.001) | -0.12  (p=0.018) | 0.36  (p<0.001) |
| **PedsQL** |  |  |  |  |  |  |  |  |  |  |
| Physical functioning | - | - | - | - | -0.52  (p<0.001) | 0.13  (p=0.014) | 0.11  (p=0.045) | 0.2  (p<0.001) | -0.19  (p<0.001) | 0.24  (p<0.001) |
| Emotional functioning | - | - | - | - | -0.16  (p=0.002) | 0.68  (p<0.001) | 0.67  (p<0.001) | 0.55  (p<0.001) | -0.26  (p<0.001) | 0.51  (p<0.001) |
| Social functioning | - | - | - | - | -0.33  (p<0.001) | -0.4  (p<0.001) | 0.5  (p<0.001) | 0.49  (p<0.001) | -0.46  (p<0.001) | 0.36  (p<0.001) |
| School functioning | - | - | - | - | -0.16  (p=0.02) | 0.27  (p<0.001) | 0.4  (p<0.001) | 0.42  (p<0.001) | -0.28  (p<0.001) | 0.26  (p<0.001) |

Table A3 known-group validity of the PROMIS-25 (six dimensions)

|  | PROMIS-25 | | | | | |  |
| --- | --- | --- | --- | --- | --- | --- | --- |
|  | Mobility | Anxiety | Depressive Symptoms | Fatigue | Peer Relationships | Pain interference | |
| **Need wheelchair** |  |  |  |  |  |  | |
| No | 25.34 (6.87) | 53.59 (9.35) | 52.66 (8.70) | 55.63 (9.73) | 42.04 (8.28) | 48.36 (9.66) | |
| Use some time | 23.45 (3.28) | 54.29 (9.45) | 53.89 (8.02) | 56.69 (7.96) | 43.70 (7.64) | 49.33 (10.53) | |
| Use all the time | 22.65 (3.05) | 53.46 (10.59) | 53.25 (8.78) | 55.77 (9.26) | 43.36 (9.47) | 52.09 (10.36) | |
| F-statistic | 9.91 | 0.25 | 0.56 | 0.47 | 1.09 | 4.1 | |
| p-value | <0.001 | 0.782 | 0.574 | 0.625 | 0.34 | 0.02 | |
| **Use of Airway Cleaning** |  |  |  |  |  |  | |
| No | 24.80 (5.92) | 53.10 (9.63) | 53.24 (8.40) | 55.22 (9.21) | 44.24 (8.50) | 49.40 (10.08) | |
| Yes | 22.67 (3.17) | 54.89 (10.50) | 53.43 (8.78) | 57.96 (8.06) | 40.75 (8.57) | 51.50 (10.33) | |
| F-statistic | 10.6 | 2.23 | 0.03 | 6.28 | 11.28 | 2.88 | |
| p-value | 0.001 | 0.136 | 0.866 | 0.013 | 0.001 | 0.091 | |
| **Scoliosis** |  |  |  |  |  |  | |
| No | 28.10 (7.63) | 52.44 (9.57) | 51.62 (9.01) | 54.27 (9.38) | 45.23 (8.88) | 47.89 (9.63) | |
| Yes | 23.19 (4.41) | 53.37 (10.01) | 53.56 (8.38) | 55.81 (8.86) | 42.81 (8.34) | 50.34 (10.13) | |
| F-statistic | 23.23 | 2.51 | 2.63 | 4.74 | 2.14 | 2.01 | |
| p-value | <0.001 | 0.083 | 0.074 | 0.009 | 0.119 | 0.136 | |
| **Hold up head without support** |  |  |  |  |  |  | |
| No | 22.01 (2.88) | 54.73 (10.45) | 55.14 (7.70) | 59.54 (9.81) | 38.32 (7.69) | 50.76 (10.42) | |
| Yes | 24.57 (5.64) | 53.39 (9.80) | 53.05 (8.56) | 55.43 (8.81) | 44.03 (8.55) | 49.81 (10.15) | |
| F-statistic | 8.13 | 0.68 | 2.22 | 7.7 | 16.61 | 0.32 | |
| p-value | 0.005 | 0.411 | 0.137 | 0.006 | <0.001 | 0.571 | |
| **Roll over to lateral position** |  |  |  |  |  |  | |
| No | 22.12 (2.84) | 54.02 (10.38) | 53.59 (8.69) | 57.36 (9.17) | 41.41 (8.43) | 51.58 (10.39) | |
| Yes | 26.31 (6.46) | 53.08 (9.37) | 53.00 (8.30) | 54.52 (8.64) | 45.25 (8.44) | 48.36 (9.73) | |
| F-statistic | 62.37 | 0.82 | 0.45 | 9,22 | 18.82 | 0.28 | |
| p-value | <0.001 | 0.366 | 0.504 | 0.003 | <0.001 | 0.002 | |
| **Sit up without support** |  |  |  |  |  |  | |
| No | 21.94 (2.91) | 54.62 (10.15) | 53.92 (8.86) | 57.26 (8.77) | 40.55 (8.26) | 51.16 (10.19) | |
| Yes | 25.58 (6.07) | 52.93 (9.68) | 52.94 (8.26) | 55.13 (9.06) | 44.97 (8.46) | 49.23 (10.11) | |
| F-statistic | 41.45 | 2.45 | 1.12 | 4.73 | 23.11 | 3.01 | |
| p-value | <0.001 | 0.118 | 0.291 | 0.03 | <0.001 | 0.083 | |
| **Four-point crawl** |  |  |  |  |  |  | |
| No | 22.94 (3.51) | 53.97 (9.83) | 53.70 (8.44) | 56.38 (8.85) | 42.89 (8.56) | 50.54 (10.37) | |
| Yes | 29.70 (8.00) | 51.79 (9.89) | 51.64 (8.50) | 53.95 (9.41) | 45.39 (8.73) | 47.42 (8.94) | |
| F-statistic | 117.34 | 2.82 | 3.42 | 4.23 | 4.87 | 5.48 | |
| p-value | <0.001 | 0.094 | 0.065 | 0.041 | 0.028 | 0.02 | |
| **Stand up without support** |  |  |  |  |  |  | |
| No | 22.79 (3.45) | 54.03 (9.94) | 53.66 (8.52) | 56.64 (9.01) | 42.48 (8.50) | 51.05 (10.38) | |
| Yes | 28.13 (7.47) | 52.27 (9.62) | 52.31 (8.34) | 53.95 (8.73) | 45.75 (8.59) | 46.98 (9.01) | |
| F-statistic | 86.48 | 2.31 | 1.86 | 6.63 | 10.75 | 12.051 | |
| p-value | <0.001 | 0.129 | 0.174 | 0.01 | 0.001 | 0.001 | |
| **Standalone without support** |  |  |  |  |  |  | |
| No | 22.89 (3.52) | 24.28 (5.45) | 53.58 (8.52) | 56.51 (9.06) | 42.54 (8.41) | 50.44 (10.46) | |
| Yes | 29.43 (7.81) | 53.84 (9.98) | 52.21 (8.32) | 53.60 (8.46) | 46.54 (8.79) | 48.00 (8.82) | |
| F-statistic | 114.62 | 1.25 | 1.58 | 6.418 | 13.48 | 3.515 | |
| p-value | <0.001 | 0.264 | 0.209 | 0.012 | <0.001 | 0.062 | |
| **Walk without support** |  |  |  |  |  |  | |
| No | 22.86 (3.44) | 53.96 (9.90) | 53.52 (8.56) | 56.46 (9.17) | 42.71 (8.47) | 50.86 (10.35) | |
| Yes | 28.91 (7.78) | 52.16 (9.71) | 52.53 (8.25) | 54.06 (8.22) | 45.60 (8.87) | 46.85 (8.96) | |
| F-statistic | 102.72 | 2.18 | 0.87 | 4.65 | 7.44 | 10.39 | |
| p-value | <0.001 | 0.141 | 0.351 | 0.032 | 0.007 | 0.001 | |
| **Walk alone without support** |  |  |  |  |  |  | |
| No | 23.02 (3.54) | 53.79 (9.86) | 53.47 (8.49) | 56.35 (9.04) | 42.89 (8.56) | 50.29 (10.46) | |
| Yes | 29.93 (8.30) | 52.41 (9.91) | 52.46 (8.49) | 53.84 (8.61) | 45.62 (8.72) | 48.23 (8.58) | |
| F-statistic | 113.5 | 1.06 | 0.76 | 4.23 | 5.45 | 2.24 | |
| p-value | <0.001 | 0.304 | 0.383 | 0.041 | 0.02 | 0.135 | |
| **Walk 10 meters on their own** |  |  |  |  |  |  | |
| No | 23.10 (3.57) | 53.70 (9.85) | 53.38 (8.49) | 56.28 (8.96) | 42.98 (8.51) | 50.28 (10.44) | |
| Yes | 30.37 (8.60) | 52.69 (10.01) | 62.83 (8.52) | 53.91 (9.04) | 45.47 (9.06) | 48.03 (8.47) | |
| F-statistic | 115.64 | 0.52 | 0.21 | 3.45 | 4.12 | 2.54 | |
| p-value | <0.001 | 0.473 | 0.649 | 0.064 | 0.043 | 0.12 | |
| **Go upstairs independently** |  |  |  |  |  |  | |
| No | 23.48 (3.94) | 53.87 (9.79) | 53.56 (8.37) | 56.28 (8.76) | 43.37 (8.45) | 50.14 (10.30) | |
| Yes | 33.44 (10.25) | 49.70 (10.20) | 50.11 (9.30) | 51.50 (10.70) | 43.49 (10.72) | 47.38 (8.31) | |
| F-statistic | 117.6 | 4.83 | 4.45 | 7.64 | 0 | 1.96 | |
| p-value | <0.001 | 0.029 | 0.036 | 0.006 | 0.947 | 0.162 | |
| **have bimanual useful function** |  |  |  |  |  |  | |
| No | 21.94 (2.77) | 54.64 (10.12) | 54.96 (8.37) | 55.43 (9.09) | 40.65 (8.31) | 52.64 (6.64) | |
| Yes | 24.87 (5.80) | 53.26 (9.80) | 52.86 (8.47) | 55.89 (9.00) | 44.08 (8.60) | 49.23 (10.20) | |
| F-statistic | 17.56 | 1.15 | 3.59 | 3.91 | 9.35 | 6.66 | |
| p-value | <0.001 | 0.285 | 0.059 | 0.049 | 0.002 | 0.01 | |
| **Raise hands over head when sitting** |  |  |  |  |  |  | |
| No | 22.47 (3.36) | 54.48 (9.99) | 54.30 (8.49) | 57.34 (8.93) | 41.52 (8.20) | 51.58 (10.16) | |
| Yes | 27.00 (6.73) | 51.97 (9.50) | 51.76 (8.27) | 53.72 (8.71) | 46.20 (8.54) | 47.42 (9.70) | |
| F-statistic | 72.25 | 6.22 | 7.96 | 14.59 | 27.38 | 15.08 | |
| p-value | <0.001 | 0.013 | 0.005 | <0.001 | <0.001 | <0.001 | |
| **Touch mouths while sitting** |  |  |  |  |  |  | |
| No | 21.96 (2.93) | 54.16 (11.48) | 52.73 (10.35) | 56.58 (10.65) | 39.34 (8.45) | 50.37 (10.74) | |
| Yes | 24.78 (5.74) | 53.40 (9.50) | 53.41 (8.03) | 55.74 (8.62) | 44.27 (8.44) | 49.82 (10.06) | |
| F-statistic | 14.85 | 0.32 | 0.35 | 0.46 | 18.22 | 0.16 | |
| p-value | <0.001 | 0.574 | 0.557 | 0.496 | <0.001 | 0.692 | |
